# Prediction of malignant lymph nodes in NSCLC by machine-learning classifiers using EBUS-TBNA and PET/CT

**DOI:** 10.1038/s41598-022-21637-y

**Published:** 2022-10-20

**Authors:** Maja Guberina, Ken Herrmann, Christoph Pöttgen, Nika Guberina, Hubertus Hautzel, Thomas Gauler, Till Ploenes, Lale Umutlu, Axel Wetter, Dirk Theegarten, Clemens Aigner, Wilfried E. E. Eberhardt, Martin Metzenmacher, Marcel Wiesweg, Martin Schuler, Rüdiger Karpf-Wissel, Alina Santiago Garcia, Kaid Darwiche, Martin Stuschke

**Affiliations:** 1 Department of Radiotherapy, West German Cancer Center, University Hospital Essen, University Duisburg-Essen, Hufelandstr. 55, 45147 Essen, Germany; 2 German Cancer Consortium (DKTK), Partner Site University Hospital Essen, Essen, Germany; 3 Department of Nuclear Medicine, West German Cancer Center, University Hospital Essen, University Duisburg-Essen, Essen, Germany; 4 Department of Thoracic Surgery and Thoracic Endoscopy, West German Cancer Center, University Medicine Essen – Ruhrlandklinik, University Hospital Essen, University Duisburg-Essen, Essen, Germany; 5 Institute of Diagnostic, Interventional Radiology and Neuroradiology, University Hospital Essen, University Duisburg-Essen, Essen, Germany; 6 Institute of Pathology, West German Cancer Center, University Hospital Essen, University Duisburg-Essen, Essen, Germany; 7 Department of Medical Oncology, West German Cancer Center, University Hospital Essen, University Duisburg-Essen, Essen, Germany; 8 Division of Thoracic Oncology, West German Cancer Center, University Medicine Essen – Ruhrlandklinik, University Duisburg-Essen, Essen, Germany; 9 Department of Pulmonary Medicine, Section of Interventional Pneumology, West German Cancer Center, University Medicine Essen – Ruhrlandklinik, University Duisburg-Essen, Essen, Germany

**Keywords:** Biomarkers, Experimental models of disease, Translational research, Cancer imaging, Cancer models, Cancer therapy, Lung cancer, Metastasis, Oncology, Mathematics and computing, Scientific data

## Abstract

Accurate determination of lymph-node (LN) metastases is a prerequisite for high precision radiotherapy. The primary aim is to characterise the performance of PET/CT-based machine-learning classifiers to predict LN-involvement by endobronchial ultrasound-guided transbronchial needle aspiration (EBUS-TBNA) in stage-III NSCLC. Prediction models for LN-positivity based on [^18^F]FDG-PET/CT features were built using logistic regression and machine-learning models random forest (RF) and multilayer perceptron neural network (MLP) for stage-III NSCLC before radiochemotherapy. A total of 675 LN-stations were sampled in 180 patients. The logistic and RF models identified SUV_max_, the short-axis LN-diameter and the echelon of the considered LN among the most important parameters for EBUS-positivity. Adjusting the sensitivity of machine-learning classifiers to that of the expert-rater of 94.5%, MLP (*P* = 0.0061) and RF models (*P* = 0.038) showed lower misclassification rates (MCR) than the standard-report, weighting false positives and false negatives equally. Increasing the sensitivity of classifiers from 94.5 to 99.3% resulted in increase of MCR from 13.3/14.5 to 29.8/34.2% for MLP/RF, respectively. PET/CT-based machine-learning classifiers can achieve a high sensitivity (94.5%) to detect EBUS-positive LNs at a low misclassification rate. As the specificity decreases rapidly above that level, a combined test of a PET/CT-based MLP/RF classifier and EBUS-TBNA is recommended for radiation target volume definition.

## Introduction

For patients with newly diagnosed locally advanced non-small cell lung cancer (NSCLC) finding the right treatment choice is often a challenge. In this context, radiotherapy represents one of the principal treatments. Improvements in systemic therapy have enabled more patients to live longer, even with metastatic disease^1,2^. This may allow and establish further treatment pathways with combined chemoradiation^3^.

Regional recurrences at five years after definitive radiochemotherapy for stage-III NSCLC were observed at cumulative incidence rates of 38.2% in the RTOG 0617 trial^4^, and 11.4% and 6.5% in the large randomised PACIFIC trial with or without durvalumab consolidation^5^. Detection of involved lymph nodes (LNs) with high sensitivity and inclusion into the radiation target volume is an important determinant for a high loco-regional tumour control after definitive radiochemotherapy.

2-deoxy-2-[^18^F]fluoro-D-glucose positron emission tomography/computed tomography (2-[^18^F]FDG-PET/CT) is the standard diagnostic procedure for definition of the radiation therapy target volume^6,7^. Positive LNs are identified and delineated by visual inspection^8,9^. Machine-learning classifiers are not readily characterised to support this routine. Endobronchial ultrasound-guided transbronchial needle aspiration (EBUS-TBNA) is recommended for mediastinal nodal staging in patients suspected of mediastinal LN involvement by PET/CT^10^. Although the use of EBUS-TBNA increased over time^11^, its additional value for target volume definition has not been analysed in prospective studies so far.

In the current work, we trained and tested the precision of machine-learning classifiers to detect EBUS-positivity from parameters systematically obtained from both PET/CT and histopathology. In addition, the role of EBUS-TBNA in increasing sensitivity with reasonable specificity (for primary tumours and LNs as well as parameters characterizing the pattern of malignant spread within the patient and histopathologic subtype) was analysed with respect to the performance of these PET/CT machine-learning classifiers.

## Materials and methods

### Study population

This work develops on our previous publications^12,13^. All 180 patients with NSCLC who consecutively presented from December/2011 to June/2018 in a centre for radiation oncology with curative treatment intent were included in the study. The Ethics Committee of the Medical Faculty of the University Duisburg-Essen approved the study (19-9056-BO). The research was performed in accordance with the Declaration of Helsinki, and with local relevant guidelines and regulations. Patients aged 18 years or older with histologically proven NSCLC were eligible. At initial diagnosis, the disease had to be potentially radically treatable, identified clinically and on the diagnostic CT-chest as stage IIIA-C (AJCC/UICC/TNM 8th-edition). Additional compulsory inclusion criteria were EBUS-TBNA sampling and [^18^F]FDG-PET/CT imaging for primary staging obtained at the same time point before treatment. Patients were excluded if they had severe systemic disease or previous tumour disease.

### [^18^F]FDG-PET/CT imaging and EBUS-TBNA

Contrast-enhanced [^18^F]FDG-PET/CT imaging was performed on the Biograph_mCT PET/CT scanner (Siemens Healthineers, Germany) after intravenous injection of 250–400 MBq 2-[^18^F]FDG ligand-complex. One expert radiologist and one nuclear medicine physician independently rated the PET/CT images and, in case of discrepancies, approved the standard report by a consensus reading. Orthogonal short and long-axis LN-diameters were also measured on CT. LNs with diffuse mediastinal infiltration were classified by an additional binary classification variable and assigned to the largest size group with a diameter of ≥ 6 cm^14^.

EBUS-TBNA was performed under general anaesthesia, and all accessible hilar and mediastinal LNs were systematically evaluated. LNs with a diameter of 5 mm or more were sampled with at least three needle passes using a 22G needle according to current guidelines^10^. EBUS-positivity was defined in this study as a positive histopathologic result from EBUS-TBNA.

Obtained tissue was placed in formalin-solution to allow the preparation of a cell block for histologic evaluation and immunocytochemical analysis. Measurements of the [^18^F]FDG-PET/CT maximum standardised uptake value (SUV_max_) were carried out in all LN-stations sampled with EBUS-TBNA.

In this dataset, there were no missing data for all parameters evaluated. The LNs were grouped into echelons 1–3 in the direction of the lymphatic drainage: from ipsilateral hilum as echelon-1, over the ipsilateral central mediastinum LN-stations 7 and ipsilateral LN-station 4 as echelon-2, to the upper ipsilateral mediastinum with LN-station 2 or to the contralateral mediastinum with LN-stations 2 and 4 or the contralateral hilum as echelon-3.

### Statistical analysis

Descriptive statistics and statistical analysis were conducted using SAS software version 9.4, SAS/STAT15.1 (SAS-Institute, Cary, NC)^15^. Several procedures, primarily FREQ, NPAR1WAY and LOGISTIC, were used to build up and compare the prognostic models. The FREQ-procedure was used to compare the performance of each classifier by the McNemar’s exact test and to test dependence of the false discovery rate (FDR) on the respective echelon by the Fisher’s exact test. The NPAR1WAY procedure performed non-parametric tests to detect location differences in the distributions of quantitative parameters.

Three types of prognostic classifiers were evaluated. The logistic model represented the first procedure to estimate the probability of EBUS-positivity in dependence on PET/CT features, LN-location and histopathology. Backward elimination at alpha = 0.05 was used for variable selection. Furthermore, the high-performance analytical procedures HPFOREST and HPNEURAL of SAS-Enterprise Miner 14.3 were used to build the random forest (RF) and multilayer perceptron neural network (MLP) models (SAS-Institute, Cary, NC)^16^. Default specifications for the RF and MLP models were adopted until otherwise stated. The MLP model internally contained four hidden layers, resulting in a minimum of false positives as the first criterion and a minimum of misclassification rates in the validation dataset as the secondary criterion, when compared to a higher or lower number of hidden layers.

The sensitivity of the logistic and MLP classifiers was calibrated by assigning weighting factors > 1 to EBUS-positive LNs compared with EBUS-negative LNs (weight = 1) in order to adjust their sensitivities to values similar to that of the expert rater. Weighting factors are not available for the RF-classifier. In addition, we adjusted the sensitivity of all classifiers to the values given in Table 4 by varying the critical cut-off level for the classifier-predicted probability of LN-involvement, at or above which the output is considered positive. Throughout this analysis, the relative weights of EBUS-positive compared to EBUS-negative LNs were used. Weighting factors were normalised so that they sum up to the sample size over the whole sample. In addition, weighting factors were utilised to compare the classification results of the different classifiers calibrated with different sensitivities. Weighting factors for the McNemar’s test can be varied independently of the factors used to calibrate the sensitivity of each classifier. Thus, the classifiers trained with different weighting factors were comparable. Furthermore, the dependence of the results on the relative weighting was analysed. Weighting factors of 9 and 20 were used to give higher weight to potential detrimental effects of a false negative classification result compared to a false positive one.

A false negative finding could lead to a regional relapse, a false positive to a slightly larger target volume with increased normal tissue toxicity.

To reduce the generalisation error, a threefold cross-validation was used. The dataset was split into three disjunctive subsets of similar size by assigning a random number to each observation. The model was trained on two subsets and the leave-out test subset was scored by the fixed model from the training set. This was repeated for all leave-out subsets, so that the entire dataset was evaluated by the model. As the MLP procedure uses a validation dataset to tune hyperparameters, nested cross validation was used^17,18^. The training datasets were further subdivided with equal probability into five random samples. The inner loop training set contained data from four of these samples and the inner loop validation set data from the remaining sample. The inner loop classifier with the highest sensitivity to detect EBUS-positive LNs in the training dataset as the first criterion and with the highest specificity as the second was used to score the leave-out test set that was unseen during classifier building.

Receiver operator characteristics (ROC) curves for all classifiers were generated using the procedure LOGISTIC and the areas under the ROC curves (AUC) were compared using a chi-square (*χ*^2^) test. All *P*-values are provided for two-sided hypotheses. The false discovery rate (FDR) is defined as the number of false positives detected by the classifier divided by the sum of false positives and true positives.

In addition, combinations of classifiers were also tested. LN-positivity is deemed to exist, if at least one of the tested classifiers, either test A or B, is positive. Under the assumption of independence of the test results the sensitivity of the combined tests (SE_ab_) is higher than the sensitivity of each individual test (SE_a_, SE_b_) according to the formula ${SE}_{ab}={SE}_{a}+{SE}_{b}-{SE}_{a}\times {SE}_{b}$. The specificity of the combined test (SP_ab_) is lower, as indicated by the formula ${SP}_{ab}={SP}_{a}\times {SP}_{b}$^19^.

### Ethics approval

Ethics committee UK Essen 19-9056-BO approved the study design, including all relevant details. We confirm that all experiments were performed in accordance with relevant regulations.

## Results

### Lymph node characteristics and patterns of spread

In total, 180 patients fulfilled the inclusion criteria. Altogether, 675 LNs were examined by EBUS-TBNA and PET/CT in all patients. The further characteristics are shown in Table 1. The distribution of LNs over the echelons were 169 (25%), 297 (44%) and 209 (31%) in LN echelon-1, -2, and-3 respectively, SUV_max_-values are available for all LN-stations and for the primary tumours. EBUS-TBNA samples were positive in 145 (86%), 126 (42%), and 20 (9.6%) at LN echelon-1, -2, and -3, respectively.

**Table 1:** Characteristics for 675 EBUS- und PET-tested lymph nodes from 180 patients with locally advanced NSCLC receiving definitive or neoadjuvant radiochemotherapy.

Lymph node characteristics	EBUS-positive nodes	EBUS-negative nodes	*P*-value
**SUV**_**max**_			< 0.0001
Median (25–75% quartile)	8.39 (5.10–13.5)	2.43 (1.85–3.20)	
**Short lymph node diameter from CT [cm]**			< 0.0001
Median (25–75% quartile)	1.75 (1.29–2.70)	0.89 (0.64–1.19)	
**Echelon**			< 0.0001
Echelon-1	145	24	
Echelon-2	126	171	
Echelon-3	20	189	
**SUV**_**max**_** primary**			0.0077
Median (25–75% quartile)	13.8 (9.8–19.0)	15.6 (11.0–20.0)	
**SUV**_**max**_** of echelon 1 lymph nodes**			< 0.0001
Median (25–75% quartile)	10.6 (6.5–16.0)	2.4 (1.8–2.9)	
**SUV**_**max**_** of echelon 2 lymph nodes**			< 0.0001
Median (25–75% quartile)	7.0 (4.2–11.8)	2.6 (2.0–3.3)	
**SUV**_**max**_** of echelon 3 lymph nodes**			< 0.0001
Median (25–75% quartile)	6.8 (4.0–11.3)	2.2 (1.8–3.1)	
**Short axis diameter of echelon 1 lymph nodes**			< 0.0001
Median (25–75% quartile)	2.04 (1.29–6.00)	0.92 (0.62–1.24)	
**Short axis diameter of echelon 2 lymph nodes**			< 0.0001
Median (25–75% quartile)	1.63 (1.29–2.15)	1.02 (0.78–1.34)	
**Short axis diameter of echelon 3 lymph nodes**			< 0.0001
Median (25–75% quartile)	1.66 (1.28–1.90)	0.79 (0.60–1.06)	
**Considered lymph node is in station 4R and primary is left-sided**			0.0026
(Yes/no)	13/278	42/342	
**Largest SUV**_**max**_** at echelon-2 for the respective patient the considered lymph node originates**			
Median (25–75% quartile)	6.5 (3.6–10.0)	4.1 (2.7–7.8)	< 0.0001
**Histology**			0.5846
(AdenoCa. vs. Non-AdenoCa.)	135/156	169/215	
**Laterality of the primary tumour the lymph node is draining**			0.0740
Right-sided	157	180	

All numbers represent lymph node counts, except in the rows with a quantitative value as SUV_max_, SUV_max_ of primary tumour and shorter diameter of EBUS-positive nodes. Continuous variables were compared by Kruskal–Wallis test. Nominal variables were compared by exact chi-square test.

**Figure 1:**
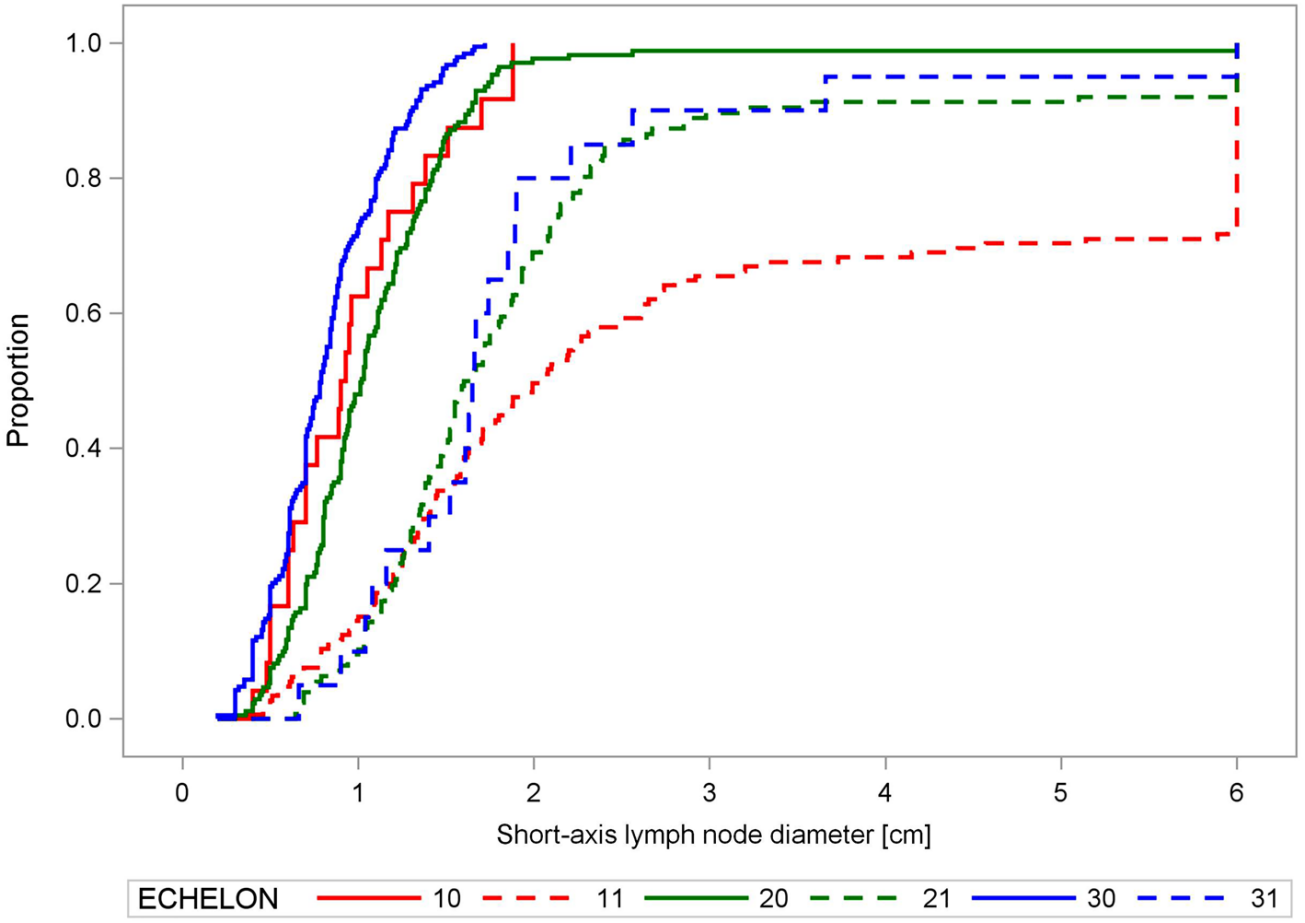
Empirical distribution functions of the short-axis lymph node diameter. Empirical distribution functions of the short-axis lymph node diameter [cm] from the computed tomograms of lymph nodes at the different echelons of the mediastinum, plotted separately for EBUS-positive and negative echelons. Group (1.0): echelon-1, EBUS-negative; Group (1.1): echelon-1, EBUS-positive; Group (2.0): echelon-2, EBUS-negative; Group (2.1): echelon-2, EBUS-positive; Group (3.0): echelon-3, EBUS-negative; Group (3.1): echelon-3, EBUS-positive. There were significant differences in the distributions of EBUS-positive and negative nodes (*P* < 0.0001, Kruskal–Wallis test).

Involved LNs were larger than uninvolved at all echelons (*P* < 0.0001; *χ*^2^ = 249.3; 1 degree of freedom (DF), Kruskal–Wallis test). The cumulative distribution of the short diameters of EBUS-positive and EBUS-negative LNs is shown in Fig. 1.

### Primary and lymph node PET-features

**Figure 2:**
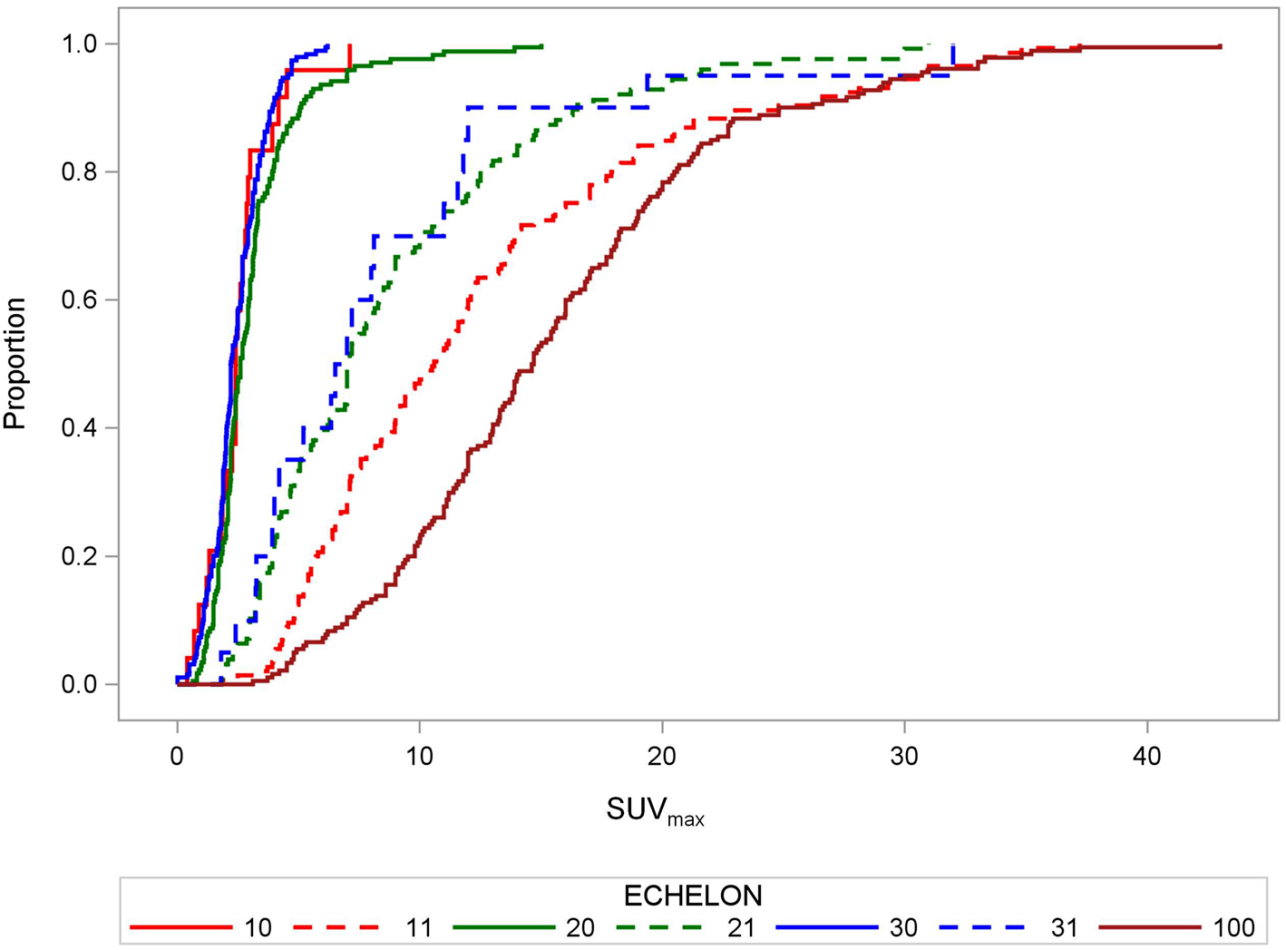
Empirical distribution functions of SUV_max_ values in lymph nodes. Empirical distribution functions of SUV_max_ in lymph nodes at the different echelons of the mediastinum, plotted separately for EBUS-positive and negative echelons. Group (1.0): echelon-1, EBUS-negative; Group (1.1): echelon-1, EBUS-positive; Group (2.0): echelon-2, EBUS-negative; Group (2.1): echelon-2, EBUS-positive; Group (3.0): echelon-3, EBUS-negative; Group (3.1): echelon-3, EBUS-positive; Group (10.0): primary tumour. The SUV_max_ for the primary tumours or EBUS-TBNA involved LNs decreased significantly from the primary tumour to echelon-1 and echelon-2 lymph nodes (*P* < 0.01, Kruskal–Wallis test), while the distributions of echelon-2 and -3 nodes did not differ.

We explored the differences and the decline of the SUV_max_ from the primary tumour to the LN echelon-1 to echelon-3. The SUV_max_ for EBUS-positive LNs decreased significantly with distance from the primary tumour toward LN echelon-1 and echelon-2, while the distributions in LN echelon-2 and -3 did not differ. The respective cumulative distribution functions are shown in Fig. 2. The median SUV_max_-values were 14.65 (5th–95th-percentile:4.85–30.35), 10.60 (5th–95th-percentile:4.05–30.70), 7.02 (5th–95th-percentile: 2.50–21.40), and 6.75 (5th–95th-percentile: 2.10–25.69) for the primary tumours and EBUS-positive echelon-1, -2, and -3 LNs, respectively. All pairwise comparisons of the SUV_max_ distributions of EBUS-positive LNs per echelon or primary tumour in Fig. 2 were significant at *P* < 0.01 (*χ*^2^ > 7.7, Kruskal–Wallis test), except between echelon-2 and echelon-3 (*P* = 0.58;*χ*^2^ = 0.39, Kruskal–Wallis test). We also found this impact of ‘distance from the primary tumour’ on SUV_max_ in an intra-patient analysis of variance for the logarithms of the SUV_max_-values (with patient and echelon as class variables) from primary to echelon-1 (*P* < 0.0001; F = 6.8 or F = 18.6, respectively;1-DF,TypeIII ANOVA F-test) and from echelon-1 to echelon-2 (*P* < 0.0001;F = 17.7;1-DF, Type-III ANOVA F-test), while the SUV_max_-values between echelon-2 vs. echelon-3 were not different (*P* = 0.27; F = 1.3; 1-DF, Type-III ANOVA F-test). The logarithms of the SUV_max_ of involved nodes were more compatible with a normal distribution than the untransformed values (*P* = 0.23, Shapiro–Wilk test on deviations of the logarithms from normal distribution). The SUV_max_ distributions of EBUS-negative LNs showed minor differences between echelons. The median SUV_max_ for EBUS-negative nodes over all echelons was 2.43 (5th–95th-percentile: 0.90–5.30).

**Figure 3:**
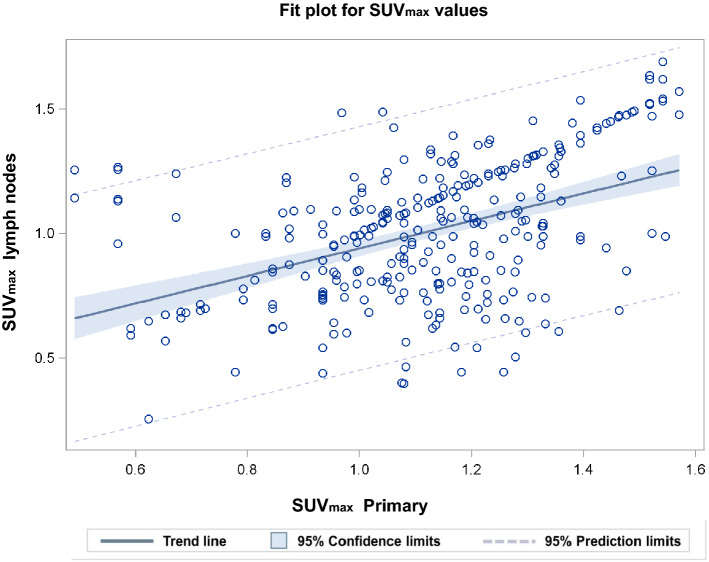
Fit plot for SUV_max_ of primary tumours and lymph nodes. The figure shows the dependence of the SUV_max_ values of EBUS-positive LNs on the SUV_max_ values of the primary tumours. Covariance plot adjusted for the echelon-effect on the SUV_max_. The R-value for the fit is 0.4496 = 0.45.

In addition, the SUV_max_ of EBUS-positive LNs were dependent on the SUV_max_ of the primary tumour. Figure 3 shows the dependence of the log (SUV_max_) of EBUS-positive LNs on the log (SUV_max_) of the primary tumours. The slope was 0.55 ± 0.06. Thereby, the log (SUV_max_) of the LNs was adjusted by an echelon-effect of − 0.328 ± 0.070 for comparison of echelon-2 with echelon-1 and of − 0.422 ± 0.137 for echelon-3 in comparison to echelon-1. SUV_max_ of EBUS-negative LNs was not related to SUV_max_ of the primary tumour (slope of 0.05 ± 0.05; *P* = 0.37, F = 0.8, Type-III ANOVA F-test).

### Multivariable logistic model to detect EBUS-positive lymph nodes

In a next step, the performance of a multivariable logistic model to predict EBUS-positivity of the respective LN was analysed based on the quantitative values of SUV_max_, CT short-axis LN-diameter (CT-SD) and parameters associated with the pattern of LN-metastases in the respective patient. The main parameters included were the continuous SUV_max_ of the primary tumour and the respective LN, the short-axis LN diameter (CT-SD) on CT together with a classification variable indicating conglomerate LN metastasis or diffuse mediastinal infiltration, the laterality of the primary tumour (left- vs. right-sided), the LN-echelon of the respective LN, the laterality of the respective LN (a variable indicating the location of the respective LN in station 4R for left-sided primary tumours^13^), the EBUS-TBNA result, and the largest SUV_max_-value in an echelon-2 LN of the respective patient. In addition, an interaction effect between SUV_max_ of the considered LN and the respective echelon were introduced. The parameter estimates from the whole dataset are shown in Table 2.

**Table 2:** Logistic model for prediction of EBUS-positivity of the respective lymph node using PET and CT information about the lymph node as well as parameters about the primary tumours and the mediastinal lymph node spread.

Covariable	Model		
	Parameter estimate (95% CI)	Wald χ^2^	*P*-value
SUV_max_	1.077 (0.665 to 1.489)	26.2, 1 DF	< 0.0001
CT-SD [cm]	1.592 (0.591 to 2.593)	9.7, 1 DF	0.0018
SUV_max_ Primary	− 0.116 (− 0.180 to − 0.051)	12.4, 1 DF	0.0004
Echelon-2 vs. Echelon-1	2.022 (0.533 to 3.512)	8.1, 2, DF	0.0178
Echelon-3 vs. Echelon-1	− 1.093 (− 3.018 to 0.832)		
SUV_max_ × Echelon-2 vs. SUV_max_ × Echelon-1	− 0.462 (− 0.868 to − 0.055)	5.1, 2 DF	0.0762
SUV_max_ × Echelon-3 vs. SUV_max_ × Echelon-1	− 0.195 (− 0.702 to − 0.312)		
Considered lymph node is in station 4R, primary is left-sided	0.810 (− 0.036 to 1.656)	3.5, 1 DF	0.0604
Largest SUV_max_ at echelon-2	− 0.061 (− 0.172 to 0.049)	1.2, 1 DF	0.2769
Lymph node conglomerate or diffuse mediastinal infiltration	− 2.001 (− 4.913 to 0.899)	1.8, 1 DF	0.1759
Histology (AdenoCa. vs. Non-AdenoCa.)	0.066 (− 0.128 to 0.249)	0.1, 1 DF	0.7488
Laterally of the primary tumour: right-sided vs. left-sided	0.312 (− 0.4369 to 1.059)	0.7, 1 DF	0.4137
Intercept	− 4.584 (− 8.651 to − 0.516)	4.9, 1 DF	0.0272

Model was fitted to the entire dataset. Parameter estimates are given with 95%-confidence intervals, χ^2^- and *P*-values and degrees of freedom (DF) are presented for each parameter. The weighting factor for EBUS-positive nodes was set ninefold higher than for EBUS-negative nodes.

*CT-SD* CT short-axis diameter of the lymph node.

To evaluate the generalisation performance of the logistic model, threefold cross-validation was applied. A weighted model was used to adjust the classifier to the same sensitivity (of 94.5%) as that of the expert rater. For the logistic model. A relative weighting factor of 9 for EBUS-positive LNs compared to EBUS-negatives resulted in a sensitivity of 94.5%, similar to that of the expert rater (Table 4). The misclassification rate was 0.2059. Increasing the sensitivity of the logistic classifier to 99.3% by lowering the cut off for predicting EBUS-positivity to Pr = 0.09 led to an increase in the misclassification rate to 0.4015 (Table 4). The SUV_max_ of the considered LN and of the primary tumour, nodal short diameter, and the echelon of the LN-localisation were the most important parameters according to the associated Wald *χ*^2^-values (Table 2).

### Machine-learning models to detect EBUS-positive lymph nodes

In comparison to the logistic model, the performance of the two machine-learning models, the MLP as well as the RF model were analysed (Table 4). All classifiers were threefold cross-validated. All parameters shown in Table 3 were used as input variables for the machine-learning models. The predictions by these models were compared with the assessment of the expert rater or a fixed threshold classifier using a SUV_max_ cut-off of 2.5 as a single criterion to announce PET-positivity. The MLP model was also adjusted to the sensitivity of the expert rater of 94.5% by weighting EBUS-positives higher than EBUS-negative LNs during training. Supplementary Figure 1 shows the diagnostic cross tabulation of the results of EBUS-TBNA and the MLP classifier.

**Table 3:** Random forest model for prediction of EBUS-positivity.

Covariable	Variable importance	
	Number of splitting rules that use this variable	Loss reduction in out of badge data
SUV_max_	1507	0.1444
Echelon-1, -2, -3	325	0.0541
CT-SD [cm]	1113	0.0398
Lymph node conglomerate or diffuse mediastinal infiltration	88	0.0003
Considered Lymph node is in station 4R and primary is left-sided	29	− 0.0002
Histology (AdenoCa. vs. Non-AdenoCa.)	147	− 0.0008
Laterality of the primary: right-sided vs. left-sided	164	− 0.0017
SUV_max_ primary	819	− 0.0239
Largest SUV_max_ at echelon-2	1259	− 0.0287

*CT-SD* CT short-axis diameter of the lymph node. Model was adapted to the whole dataset.

In addition, the precision of the logistic, the RF or the MLP models were analysed at a very high sensitivity of 97.9% and 99.3%, by lowering the cut-off predicted probability at which EBUS-positivity was announced. Because the RF model does not support weighting factors, its sensitivity was adjusted to a similar sensitivity as the MLP model by lowering the critical cut-off values for the predicted probability of EBUS-positivity to Pr = 0.221, 0.08 or 0.05. The lowest predicted probability of an EBUS-positive LN was Pr = 0.0003 according to this model. Similar to the logistic model, the SUV_max_, the considered LN-echelon and the short-axis LN diameter were identified as the most important variables by the RF model according to the loss reduction criterion. However, the SUV_max_ of the primary tumour appeared less important. The variable importance is proportional to the sum of the reduction of the node impurity at the nodes where each variable splits (Table 3).

Table 4 depicts the misclassification rates (MCRs) and the number of false negative LNs compared to EBUS-TBNA for all cross-validated classifiers, as well as the results of the expert rater and a fixed threshold classifier with a SUV_max_ cut-off of 2.5 as a single criterion. At the same sensitivity of 94.5% as the expert rater, the MCR of the RF model was slightly (*P* = 0.038; McNemar’s test) and that of the MLP markedly better (*P* = 0.0061; McNemar’s test), than that of the standard report, giving false negatives the same weight as false positives within the McNemar’s test. In addition, weighting false positives and negatives equally, the logistic model at a sensitivity of 94.5% showed a similar performance (*P* = 0.066, McNemar’s test). The fixed threshold model at a cut-off of 2.5 was significantly worse with a higher misclassification rate (*P* < 0.0001, McNemar’s test) than the standard report. At higher sensitivities of 97.9%, the logistic, MLP and RF classifier ranked better than the nuclear medicine rater but only if false negatives received a 20-fold higher weight than false positives (*P* < 0.05, McNemar’s tests for weight_MCN_ = 20, Table 4). The same applies for the MLP and RF classifier at the sensitivity of 99.3% (Table 4). The reduced numbers of false negatives over-compensate the increase in the misclassification rate for the latter classifiers (Table 4).

**Table 4:** Performance of the logistic model and two machine-learning models in comparison to the standard report.

Model/classifier	Adjusted sensitivity	MCR	False negatives	McNemar’s test for comparison of the row heading classifier with standard report by expert rater
Logistic model, cross-validated	Sensitivity = 94.5% by Weighting factor = 9 Cut-off Pr = 0.50	0.2059	16	0.0663 (weight_MCN_ = 1))
Logistic model, cross-validated	Sensitivity = 97.9% by Weighting factor = 9 Cut-off Pr = 0.30	0.2756	6	0.7179 (weight_MCN_ = 9) 0.0447 (weight_MCN_ = 20)
Logistic model, cross-validated	Sensitivity = 99.3% by Weighting factor = 9 Cut-off Pr = 0.09	0.4015	2	0.2853 (weight_MCN_ = 9) 0.0979 (weight_MCN_ = 20)
MLP, cross-validated	Sensitivity = 94.5% by Weighting factor = 3 Cut-off Pr = 0.48	0.1333	16	0.0061 (weight_MCN_ = 1)
MLP, cross-validated	Sensitivity = 97.9% Weighting factor = 3 Cut-off Pr = 0.15	0.2133	6	0.1054 (weight_MCN_ = 9) 0.0088 (weight_MCN_ = 20)
MLP, cross-validated	Sensitivity = 99.3% by Weighting factor = 3 Cut-off Pr = 0.05	0.2978	2	0.4203 (weight_MCN_ = 9) 0.0052 (weight_MCN_ = 20)
RF, cross-validated	Sensitivity = 94.5% by Cut-off Pr = 0.221	0.1452	16	0.0375 (weight_MCN_ = 1)
RF, cross-validated	Sensitivity = 97.9% by Cut-off Pr = 0.08	0.2667	6	0.5966 (weight_MCN_ = 9) 0.0432 (weight_MCN_ = 20)
RF, cross-validated	Sensitivity = 99.3% by Cut-off Pr = 0.04	0.3422	2	0.9790 (weight_MCN_ = 9) 0.0270 (weight_MCN_ = 20)
Standard report by expert rater		0.1748	16	
SUV_max_ = 2.5 as fixed cut-off		0.2919	9	< 0.0001 (weight_MCN_ = 1) 0.4395 (weight_MCN_ = 9)

False negatives: number of EBUS-positive lymph nodes estimated to be EBUS-negative by the model. Weighting factors for EBUS-positive nodes compared to EBUS-negatives (weight = 1) were introduced to adjust the sensitivity of the MLP or logistic model similar to that of the specialist rater of 94.5% at a classification threshold of about 0.5. In addition, the cut-off probability for the classifier used to announce PET-positivity was chosen to adjust the sensitivity also to higher values of 97.9 and 99.3% as indicated in column 2. The McNemar’s test was used to compare the performance of the classifier in the respective row of the table with the standard report by the specialist rater.

As the clinical consequences of false positives and negatives are different, a weighted McNemar’s test was used and the weighting factors for EBUS positive lymph nodes using this test (weight_MCN_) relative to those of EBUS negative nodes are indicated. Error rate: number of misclassifications over the 675 assessed lymph nodes.

*MCR* misclassification rate, *MLP* multilayer perceptron neural network; *RF* Random forest.

**Figure 4:**
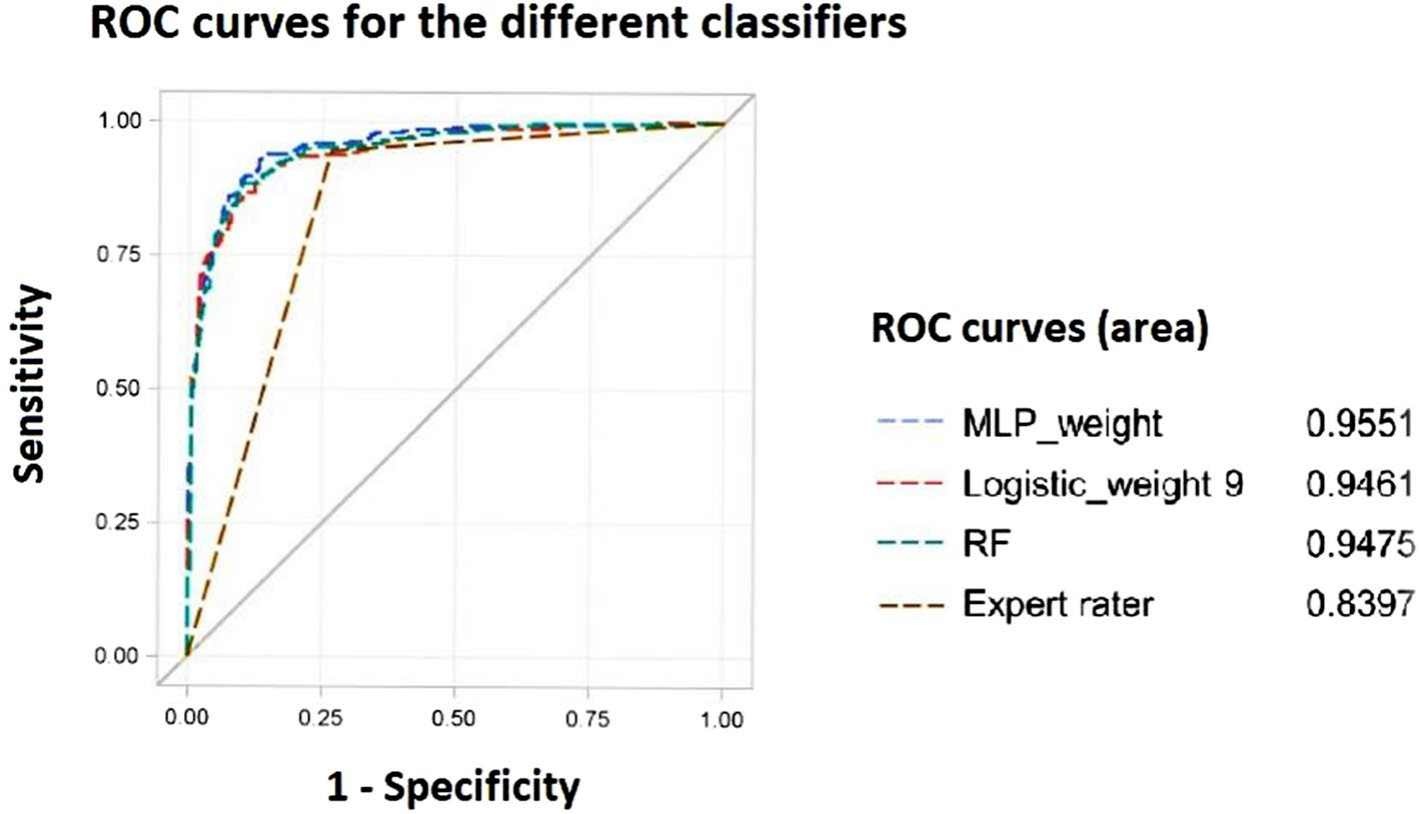
ROC curves for the different classifiers. The figure shows the receiver operator characteristic (ROC) curves for comparing the performance of the cross-validated multilayer perceptron neural network (MLP), random forest (RF) and logistic regression models with the expert rater. The logistic regression model was trained with a weighting factor 9 and the MLP model with a weighting factor of 3.06 for false negatives to achieve the same sensitivity as the expert rater at a cut-off point for the estimated probability of EBUS-positivity of 0.5. In addition, the ROC curve for the MLP trained with a weighting factor of 10 for EBUS-positives is shown.

Figure 4 shows the ROC curves for the different cross-validated classifiers compared to the results of the expert rater. The MLP classifier ranked best followed by the RF and the logistic model (both *P* = 0.034 *and P* = 0.0063 in comparison to MLP at 94.5%, respectively), while the latter two classifiers had similar areas under curve (*P* = 0.15; *χ*^2^ = 0.70 (1-DF). Area under curves stayed together with their 95% Wald confidence intervals 0.9551 (95% CI 0.9438–0.9663), 0.9475 (95% CI 0.9349–0.9600), and 0.9461 (95% CI 0.9339–0.952) above 0.93 for all cross-validated machine-learning classifiers and the logistic regression models shown in Table 4.

As some patients of this study received EBUS-TBNA prior to PET/CT, we analysed the influence of the sequence of both procedures on the results by the expert rater. A total of 56 LNs were biopsied 4 or more days prior to PET/CT, 154 nodes between 3 and 1 day prior and the remaining 465 nodes were biopsied after PET/CT. Neither sensitivity nor specificity of the standard report by the expert rater to predict EBUS-positivity was dependent on the sequence of the test (PET/CT and EBUS). Sensitivity of PET/CT performed prior to or after EBUS-TBNA was 95.1% (95% CI 91.2–97.6%) and 93.1% (95% CI 85.6–97.4%) (*P* = 1.00; Fisher’s exact test). In addition, the sensitivity of PET/CT performed 1–3 days after EBUS-TBNA was similar with 96.9% (95% CI 89.2–99.6%). Specificity of PET/CT performed prior to or after EBUS-TBNA was 73.6% (95% CI67.8–78.9%) and 73.2% (95% CI 64.4–80.1%) (*P* = 0.58, Fisher’s exact test) and the specificity of PET/CT performed 1–3 days after EBUS-TBNA remained also unchanged with 72.2% (95% CI 61.8–81.2%).

One finding from our previous work was that the FDR by the expert rater increases from echelon-1 to echelon-3^12^. For both machine-learning classifiers at the high overall sensitivity of 99.3%, the FDR increases significantly from 13.6 over 37.4 to 70.3% for the RF and from 11.6%, over 50.4% to 74.3% for the MLP (*P* ≤ 0.001, Fisher’s exact test for all pairwise comparisons). At echelon-3, the FDR at a sensitivity of 94.5% was lowest from the MLP classifier.

### Machine-learning models to detect LN-involvement augmented by EBUS-TBNA

Because MCR and FDR increase sharply when the sensitivity of the classifiers exceeds 94.5%, we analysed some combined tests for predicting the regional pattern of spread of LN metastases based on a PET/CT MLP classifier performed at a sensitivity of 94.5% on one hand and on the RF classifier or the expert rater on the other. The result of a combined test is deemed positive, if one or both of the individual tests are positive. Table 5 shows that the overall sensitivity of the combined test of MLP and RF models increased to 96.6% as well as the sensitivity of the combined test of MLP and the expert rater to 96.9%. Both values are less than the sensitivity of 99.7%, expected due to the independence of the test results. Fourty-four percent of the EBUS-TBNA positive and expert rater negative LNs became positive by the combined test at the price that per additionally discovered EBUS-positive LNs four additional uninvolved LNs became positive.

**Table 5:** Determination of misclassification and false discovery rate in echelon-1 to -3 with each test procedure.

Model/classifier	Echelon-1 No of LN = 169 No of EBUS + LN = 145			Echelon-2 No of LN = 297 No of EBUS + LN = 126			Echelon-3 No of LN = 209 No of EBUS + LN = 20		
	Misclassifi-cation error rate (MER)	False negatives	FDR	Misclassifi-cation error rate (MER)	False negatives	FDR	Misclassifi-cation error rate (MER)	False negatives	FDR
Logistic model, cross-validated at sensitivity = 94.5%	0.0769	1	0.077	0.3434	8	0.443	0.1148	7	0.567
MLP at sensitivity = 99.3%, cross-validated	0.1124	0	0.116	0.4310	0	0.504	0.2584	2	0.743
MLP at sensitivity = 97.9%, cross-validated	0.1006	1	0.100	0.3230	2	0.431	0.1388	3	0.605
MLP at sensitivity = 94.5%, cross-validated	0.0769	3	0.066	0.2189	8	0.326	0.0574	5	0.318
RF at sensitivity = 99.3%, cross-validated	0.1361	0	0.137	0.3737	5	0.467	0.2201	1	0.703
RF at sensitivity = 94.5%, cross-validated	0.0592	0	0.065	0.2256	12	0.325	0.1005	4	0.515
Cut-off SUV_max_ = 2.5 as a single fixed criterion	0.0710	1	0.071	0.3333	6	0.444	0.4115	2	0.824
Specialist reader	0.0533	1	0.053	0.2290	12	0.329	0.1962	3	0.691
Combined test, MLP at sensitivity = 94.5% or RF at sensitivity 94.5%, cross-validated	0.0710	0	0.076	0.2492	6	0.362	0.1053	4	0.529
Combined test, MLP at sensitivity = 94.5% or specialist reader	0.0651	0	0.071	0.2896	6	0.400	0.2010	3	0.696

*MLP* multilayer perceptron neural network, *RF* Random forest, *LN* lymph node, *FDR* false discovery rate.

With regard to a combined test of EBUS-TBNA and PET/CT, it is advantageous to make use of the high specificity of EBUS-TBNA of about 100%. To demonstrate this, a sensitivity of EBUS-TBNA of 85% is assumed to detect truly involved LNs as well as independence of both tests, EBUS-TBNA and the PET-based MLP classifier. This results in a prevalence of 342 truly involved LNs. The combined test of the MLP classifier with a sensitivity of 94.5%, together with the 16 EBUS-TBNA positives that are MLP classifier negative results in a miss of 2.8 false negative nodes. As the total number of positives by the MLP classifier was 349 (Table 4), a total of 12.6 misclassifications are observed. This result can be compared with the MLP classifier alone at a sensitivity of 99.3%. At a prevalence of 342 truly involved LNs, this classifier misses 2.4 false negative nodes and results in 150.8 misclassifications. Comparing the combined test with the MLP classifier at a sensitivity of 99.3%, superiority of the combined test can be claimed using weighting factors of 9 and 20 (*P* < 0.0005, McNemar’s tests).

## Discussion

Meta-analysis of clinical studies revealed overall sensitivities of 77.4% and specificities of 90.1% of PET/CT for mediastinal staging (N2/N3-mediastinal involvement) per patient according to qualitative visual inspection^20^. The 95%-confidence intervals (95% CI) of the sensitivities and specificities from different studies were moderate with 65.3–86.1% and 85.3–93.5%, respectively. Therefore, expert raters placed considerable attention on a high specificity and thus on the avoidance of false positive findings with the possible consequence of an unjustified denial of surgery for the patient. EBUS-TBNA is generally recommended for histopathological proof of patients with PET-positive LNs prior to therapy or if there is an increased risk of mediastinal involvement^10^. However, for radiation therapy planning, the consequences of a false negative finding that could lead to a spatial miss of a LN-metastasis and thus recurrence, are potentially higher than a false positive finding with a slight extension of the target volume. However, radiotherapy to the whole mediastinum should be avoided, as a larger randomised trial showed that PET-based radiotherapy to the mediastinum according to the identification of involved LNs by a radiation oncologist in consensus with a specialist nuclear-medicine expert is not inferior to the prophylactic mediastinal irradiation at the loco-regional control endpoint^8^. In their meta-analysis, Gould et al. found higher sensitivities of ^18^F-FDG PET in patients with enlarged LNs on CT than in those without^21^. As the summary ROC curves for these groups of patients were the same, these data revealed the dependence of the operating point on the ROC curve on the presence of enlarged LNs. This study also found a similar sensitivity of PET in studies scoring involvement on a per LN-basis than in studies using mediastinal involvement on a per patient basis (yes_or_no) as the target. A cut-point for SUV_max_ within the LN as a quantitative criterion for PET-positivity has the advantage that the sensitivity on the ROC-curve can be preselected by this quantitative measure.

Schmidt-Hansen et al. found a similar sensitivity and specificity of PET/CT to predict mediastinal involvement on a per patient basis in studies using a predefined SUV_max_ cut-off point of 2.5 for a positive LN than in the studies using visual inspection of the activity over background of the LNs^20^. Yang et al. analysed sensitivity and specificity of PET/CT per LN using either the activity of the mediastinal blood pool (MBP) or a SUV_max_ of 3.9 above as cut-points for PET-positivity and compared these results with histopathology of EBUS-TBNA or resection specimens^22^. Sensitivities were 97.4% and 90.9% and specificities were 35.8% and 61.9% using the first or second of the aforementioned criteria. As sensitivities of more than 95% are achievable, specificities of about 36% are rather low. Nguyen et al. also found a sensitivity of 90% at a SUV_max_ cut-point of 3.9 within the LN^23^.

In the present study, the sensitivities of 94.5% and 96.9% and specificities of 73.4% and 51.0% were achieved by the expert rater or using an SUV_max_ cut-point of 2.5 for the considered LN. As found in another study, specificity of the expert rater did not depend on the sequence of EBUS-TBNA and PET/CT to a detectable extent^24^.

Furthermore, it was shown here, that the SUV_max_ of an EBUS-TBNA positive LN was dependent on the SUV_max_ of the primary tumours and decreased from the primary tumour to echelon-1 and from echelon-1 to echelon-2 or echelon-3 in an intra-patient analysis. The SUV_max_ seems to depend on the particular LN-echelon. Possible reasons for the SUV_max_ decline is a smaller cell density at more distant nodes, a smaller FDG-accumulation per tumour cell in more distant nodes, and a smaller diameter of involved nodes at echelon-2 and -3 in comparison to echelon leading to smaller signal recovery coefficients. Accordingly, Li et al. found that the false positive rate (FPR) for detection of the mediastinal LN involvement by PET increased in tumours with SUV_max_ ≤ 4.0 using a SUV_max_ cut-point of 2.5 as criterion^25^. Fixed threshold criteria do not consider a systematic dependence of SUV_max_ on distance from the primary tumour, whereas machine-learning classifiers or multivariable regression models can do so.

Vesselle et al. used a multilayer perceptron neural network to predict mediastinal involvement from basic diagnostic PET and CT parameters on a per patient basis^26^. A higher accuracy on the test sets was achieved by the MLP (87.3%) than by the expert rater (73.5%). For comparison, the accuracy of MLP (weight = 3.06) and the expert rater in the present study were 88.0% and 82.5%, respectively. Wang et al., found classical machine-learning methods to predict LN positivity in LN-based surgical specimens. Input parameters were diagnostic parameters for the LNs from PET/CT, such as LN short diameter or SUV_max_ and related parameters, as well as 82 radiomic texture features^27^. While the radiomic texture features did not improve prediction, the accuracy ranged from 82.7 to 85.1%, and the various classifiers using diagnostic PET/CT parameters achieved sensitivities of 77.1% to 85.7%. Accuracy and sensitivity of the expert rater were 81.6% and 72.9%, respectively. A deep convolutional network directly analysing the image patches around the LNs, showed similar results as classical machine-learning methods using a set of diagnostic features from PET/CT. Yoo et al. analysed PET/CT features of LNs from lung cancer patients with or without distant metastases using machine-learning classifiers to predict the histopathologic results after surgery or minimally invasive procedures^28^. The prevalence of histopathologic LN positivity was with 61% higher in their study than in the present study with 43%. The multiplicity of analysed LNs per patient was 1.36 in that study, much smaller than in the present study with 3.75, so that the index lesion per patient was analysed in the former while the intra-patient spread was analysed in the present study. The areas under ROC curves by the machine-learning algorithm was about 0.85 and therefore smaller than those found in the present study that uses also location parameters of the LNs. In both studies, SUV_max_ was a dependent LN variable with the highest importance. They found similar accuracies of the machine-learning classifier using PET/CT features and the expert rater. Sibille and Seifert trained a deep convolutional neural network to directly localise and classify PET/CT foci from PET/CT image datasets of lymphoma and NSCLC-patients. Nuclear-medicine experts’ readings were the reference standard in that study. For lung cancer a classification sensitivity of 87.1% and a specificity of 99.1% were found in comparison to the expert rater^29^.

In the present study, we used classical diagnostic features from PET/CT together with localisation parameters of the LNs as well as the primary tumour, parameters related to the spread of LNs to echelon-2 in the mediastinum and histopathology to serve as input and set the machine-learning classifiers.

One method to compare the performance of different classifiers is to fix sensitivity and take specificity as the measure of performance^30,31^. We compared the three classifiers logistic, MLP, and RF at three sensitivities, 94.5% as obtained by the nuclear medicine specialist, and even higher sensitivities of 97.9% and 99.3%. This was achieved by variation of the classification threshold for each test. In addition, as the logistic and the RF models allow for differing weighting factors placed on the prediction error for EBUS-positives or negatives nodes, giving different weights to false positive and negative predictions. These weights were adjusted to result in a classifier sensitivity of 94.5% at a classification threshold of the classifier about 0.5. At the same sensitivity, the MLP classifier generally shows the highest specificity. In addition, all the classifiers were compared with the performance of the nuclear medicine specialist using the McNemar’s test. At the same sensitivity of 94.5%, the MLP classifier ranked best while the logistic model ranked worse. At higher sensitivities, the comparison of the machine learning classifier with the nuclear medicine specialist depends on the relative importance given to false positive and false negative classifications. As the consequence of a false negative classification, i.e. a relapse, might be much more adverse than that of a false positive result, i.e. enlargement of the target volume, importance factors (weight_MCN_) were introduced given the relative weight of false EBUS negative results in comparison to false positives^32^. If false negative results count 20 fold more than false negatives the machine learning classifier at very high sensitivities of 97.9 and 99.3% performed consistently better than the standard reporting procedure.

The influence of the echelon on which the LN resides on the sensitivity of the PET/CT readings by the specialist experts was shown in our previous study on this same dataset^12^. The sensitivity decreased from 99.3% on echelon-1 to 90.5% on echelon-2 and to 85.0% on echelon-3. On a per patient basis, EBUS-positivity at echelon-3 depended on pattern of spread of the tumour^13^. The present study shows that machine-learning methods may assist the expert rater at a high sensitivity of 94.5%. Especially the MCR at echelon-2 and -3 could be substantially reduced by the MLP classifier compared to the standard reporting procedure with the same sensitivity. Increasing the sensitivity beyond 94.5%, the MCR of the machine-learning classifiers increased rapidly, with the smallest increase for the MLP classifier. Therefore, a suitable way to combine the results of a machine-learning classifier with EBUS-TBNA results, which are available in the majority of thoracic oncology centres but are not systematically used to define the target volume for radiation therapy, is important.

The meta-analysis by Leong et al. showed a pooled sensitivity of EBUS-TBNA of 49% for detecting unsuspected N2/N3 mediastinal involvement in a PET–negative mediastinum with a specificity of 100%^33^. Similar characteristics were found in the meta-analysis conducted by El-Osta 2018^34^. The pooled prevalence of N2/N3 in these studies underlying the meta-analyses was 13–15%. However, with a higher prevalence of mediastinal metastases of about 34%, the sensitivity of EBUS-TBNA in combination with EUS-FNA to detect N2/N3 mediastinal metastases can be markedly higher and was found to be about 86%^35^. Here, we propose a combined test using a machine-learning classifier based on diagnostic PET/CT parameters and the results of systematic EBUS-sampling also from PET-negative LNs to bring the sensitivity of the PET/CT classifier above 94.5%, while maintaining specificity. Assuming that both tests are independent, the sensitivity of the combined test yielding a positive result increases from 94.5% of the MLP-classifier alone to 99.2% in the case of either MLP PET/CT or EBUS-TBNA positivity when the sensitivity of EBUS-TBNA ranges from 85%^19^. Since the MCR of PET/CT-based machine-learning classifiers increases rapidly above a sensitivity of 94.5% according to this study, a combined test of PET/CT-based machine-learning classifiers adjusted to sensitivity of 94.5%, in combination with a systematic EBUS-TBNA of PET-negative LN stations can be recommended for defining the target volume for radiotherapy of stage-III NSCLC to achieve sensitivities for LN-metastases detection above 94.5%. This combined assay takes advantage of the high specificity of EBUS-TBNA and uses the machine-learning to improve the specificity of the PET/CT results at a high sensitivity of the expert rater. In addition, the risk of missed LNs due to inaccessibility by EBUS-TBNA or a varying negative predictive value of EBUS-TBNA observed in several studies is mitigated^36–39^.

With the limitation of all prescriptions, target volumes based on algorithms should be controlled according to high known standards, i.e. in prospective studies^40–42^.

## Conclusion

PET/CT based machine-learning classifiers demonstrate the potential to reduce the MCR compared to the standard report. Because misclassification increases substantially at higher sensitivities, a combined test of a PET/CT-based machine-learning classifier with a systematic EBUS-TBNA of PET-negative LN-stations is recommended. The classifiers can support the specialist to increase sensitivity. This dual test performed better than machine-learning classifiers alone. Combined, classifiers based on [^18^F]FDG-PET/CT features and EBUS-TBNA prove to be valuable instruments for determining the regional pattern of nodal spread and radiation treatment planning in lung cancer.

## Supplementary Information

Supplementary Legends.

Supplementary Figure 1.

## Data Availability

All data generated and analysed during this study are included in this published article (Supplementary Information can be received from the corresponding author).

## References

[1] Hellmann MD, Paz-Ares L, Bernabe Caro R (2019). Nivolumab plus ipilimumab in advanced non-small-cell lung cancer. N. Engl. J. Med..

[2] Hui R, Ozguroglu M, Villegas A (2019). Patient-reported outcomes with durvalumab after chemoradiotherapy in stage III, unresectable non-small-cell lung cancer (PACIFIC): A randomised, controlled, phase 3 study. Lancet Oncol..

[3] Palma DA, Olson R, Harrow S (2019). Stereotactic ablative radiotherapy versus standard of care palliative treatment in patients with oligometastatic cancers (SABR-COMET): A randomised, phase 2, open-label trial. Lancet.

[4] Bradley JD, Hu C, Komaki RR (2020). Long-term results of NRG oncology RTOG 0617: Standard- versus high-dose chemoradiotherapy with or without cetuximab for unresectable stage III non-small-cell lung cancer. J. Clin. Oncol..

[5] Antonia SJ, Villegas A, Daniel D (2018). Overall survival with durvalumab after chemoradiotherapy in stage III NSCLC. N. Engl. J. Med..

[6] Nestle U, De Ruysscher D, Ricardi U (2018). ESTRO ACROP guidelines for target volume definition in the treatment of locally advanced non-small cell lung cancer. Radiother. Oncol..

[7] De Ruysscher D, Faivre-Finn C, Moeller D (2017). European Organization for Research and Treatment of Cancer (EORTC) recommendations for planning and delivery of high-dose, high precision radiotherapy for lung cancer. Radiother. Oncol..

[8] Nestle U, Schimek-Jasch T, Kremp S (2020). Imaging-based target volume reduction in chemoradiotherapy for locally advanced non-small-cell lung cancer (PET-Plan): A multicentre, open-label, randomised, controlled trial. Lancet Oncol..

[9] Lapa C, Nestle U, Albert NL (2021). Value of PET imaging for radiation therapy. Strahlenther. Onkol..

[10] Vilmann P, Clementsen PF, Colella S (2015). Combined endobronchial and esophageal endosonography for the diagnosis and staging of lung cancer: European Society of Gastrointestinal Endoscopy (ESGE) Guideline, in cooperation with the European Respiratory Society (ERS) and the European Society of Thoracic Surgeons (ESTS). Endoscopy.

[11] Ost DE, Niu J, Zhao H, Grosu H, Giordano SH (2019). Quality gaps and comparative effectiveness in lung cancer staging and diagnosis. Chest.

[12] Guberina M, Darwiche K, Hautzel H (2021). Impact of EBUS-TBNA in addition to [(18)F]FDG-PET/CT imaging on target volume definition for radiochemotherapy in stage III NSCLC. Eur. J. Nucl. Med. Mol. Imaging.

[13] Guberina M, Darwiche K, Hautzel H (2021). Patterns of nodal spread in stage III NSCLC: Importance of EBUS-TBNA and (18)F-FDG PET/CT for radiotherapy target volume definition. Radiat. Oncol..

[14] Silvestri GA, Gonzalez AV, Jantz MA (2013). Methods for staging non-small cell lung cancer: Diagnosis and management of lung cancer, 3rd ed: American College of Chest Physicians evidence-based clinical practice guidelines. Chest.

[15] *SAS Release 9.4, SAS/STAT 15.1 User's Guide* (SAS Institute, Inc., 2018).

[16] SAS Institute Inc. *SAS Enterprise Miner 14.3*. https://documentation.sas.com/doc/de/pgmsascdc/9.4_3.2/emhpprcref/titlepage.htm (SAS Institute Inc., 2018). Accessed 5 october 2022.

[17] Stone M (1974). Cross-validatory choice and assessment of statistical predictions. J. R. Stat. Soc.: Ser. B (Methodol.).

[18] Stone M (1974). Cross-validatory choice and assessment of statistical predictions. J. R. Stat. Soc. Ser. B (Methodolol.).

[19] Weinstein S, Obuchowski NA, Lieber ML (2005). Clinical evaluation of diagnostic tests. Am. J. Roentgenol..

[20] Schmidt-Hansen M, Baldwin DR, Hasler E, Zamora J, Abraira V, Roqué IFM (2014). PET-CT for assessing mediastinal lymph node involvement in patients with suspected resectable non-small cell lung cancer. Cochrane Database Syst. Rev..

[21] Gould MK, Kuschner WG, Rydzak CE (2003). Test performance of positron emission tomography and computed tomography for mediastinal staging in patients with non-small-cell lung cancer: A meta-analysis. Ann. Intern. Med..

[22] Yang DD, Mirvis E, Goldring J, Patel ARC, Wagner T (2019). Improving diagnostic performance of (18)F-FDG-PET/CT for assessment of regional nodal involvement in non-small cell lung cancer. Clin. Radiol..

[23] Nguyen P, Bhatt M, Bashirzadeh F (2015). Comparison of objective criteria and expert visual interpretation to classify benign and malignant hilar and mediastinal nodes on 18-F FDG PET/CT. Respirology (Carlton).

[24] Ramsahai JM, Molnar C, Lou L (2020). Does prior mediastinal lymph node aspiration contribute to false-positive positron emission tomography-computed tomography?. ERJ Open Res..

[25] Li S, Zheng Q, Ma Y (2013). Implications of false negative and false positive diagnosis in lymph node staging of NSCLC by means of ^18^F-FDG PET/CT. PLoS ONE.

[26] Vesselle H, Turcotte E, Wiens L, Haynor D (2003). Application of a neural network to improve nodal staging accuracy with 18F-FDG PET in non-small cell lung cancer. J. Nucl. Med..

[27] Wang H, Zhou Z, Li Y (2017). Comparison of machine learning methods for classifying mediastinal lymph node metastasis of non-small cell lung cancer from (18)F-FDG PET/CT images. EJNMMI Res..

[28] Yoo J, Cheon M, Park YJ (2021). Machine learning-based diagnostic method of pre-therapeutic (18)F-FDG PET/CT for evaluating mediastinal lymph nodes in non-small cell lung cancer. Eur. Radiol..

[29] Sibille, L. & Seifert, R. (*18)F-FDG PET/CT Uptake Classification in Lymphoma and Lung Cancer by Using Deep Convolutional Neural Networks*, Vol. 294, 445–452 (2020). 10.1148/radiol.2019191114 31821122

[30] Metz CE (1978). Basic principles of ROC analysis. Semin. Nucl. Med..

[31] Hand DJ (2010). Evaluating diagnostic tests: The area under the ROC curve and the balance of errors. Stat. Med..

[32] Rücker G, Schumacher M (2010). Summary ROC curve based on a weighted Youden index for selecting an optimal cutpoint in meta-analysis of diagnostic accuracy. Stat. Med..

[33] Leong TL, Loveland PM, Gorelik A, Irving L, Steinfort DP (2019). Preoperative staging by EBUS in cN0/N1 lung cancer: Systematic review and meta-analysis. J. Bronchol. Interv. Pulmonol..

[34] El-Osta H, Jani P, Mansour A, Rascoe P, Jafri S (2018). Endobronchial ultrasound for nodal staging of patients with non-small-cell lung cancer with radiologically normal mediastinum. A meta-analysis. Ann. Am. Thorac. Soc..

[35] Korevaar DA, Crombag LM, Cohen JF, Spijker R, Bossuyt PM, Annema JT (2016). Added value of combined endobronchial and oesophageal endosonography for mediastinal nodal staging in lung cancer: A systematic review and meta-analysis. Lancet Respir. Med..

[36] Rintoul RC, Tournoy KG, El Daly H (2009). EBUS-TBNA for the clarification of PET positive intra-thoracic lymph nodes—An international multi-centre experience. J. Thorac. Oncol..

[37] Adams K, Shah PL, Edmonds L, Lim E (2009). Test performance of endobronchial ultrasound and transbronchial needle aspiration biopsy for mediastinal staging in patients with lung cancer: Systematic review and meta-analysis. Thorax.

[38] Fuso L, Varone F, Magnini D (2017). Influence of the learning effect on the diagnostic yield of endobronchial ultrasound-guided transbronchial needle aspiration of mediastinal and hilar lymph nodes. J. Bronchol. Interv. Pulmonol..

[39] Crombag LMM, Dooms C, Stigt JA (2019). Systematic and combined endosonographic staging of lung cancer (SCORE study). Eur. Respir. J..

[40] *Proposed Regulatory Framework for Modifications to Artificial Intelligence/Machine Learning (AI/ML)-Based Software as a Medical Device (SaMD)—Discussion Paper and Request for Feedback*. https://www.fda.gov/media/122535/download. (US Food and Drug Administration (FDA), 2019). Accessed 5 october 2022.

[41] Wang F, Casalino LP, Khullar D (2019). Deep learning in medicine-promise, progress, and challenges. JAMA Intern. Med..

[42] Beam AL, Kohane IS (2018). Big data and machine learning in health care. JAMA.

