# Supplementary material for: Prediction of malignant lymph nodes in NSCLC by machine-learning classifiers using EBUS-TBNA and PET/CT
Source: Sci Rep. 2022 Oct 20;12:17511. doi: 10.1038/s41598-022-21637-y (PMC9584941; doi:10.1038/s41598-022-21637-y)
Supplement: Supplementary file 1 — Supplementary Legends. [file 41598_2022_21637_MOESM1_ESM.docx]

**Fig 1 Suppl. Flow chart:** Consecutive patients that match the inclusion criteria of this retrospective trial, i.e. conduct of both EBUS/TBNA and PET/CT before start of treatment in patients with locally advanced non-small cell lung cancer receiving definitive or neoadjuvant radiochemotherapy at the West German Cancer Center. The chart shows diagnostic cross tabulation of the results of EBUS-TBNA (EBUS+ vs. EBUS-) with the results of the cross-validated PET-based multilayer perceptron neural network classifier (MLP+, MLP-) at a sensitivity of 94.5% per lymph node (LN) and per patient. Agreement of both tests in all sampled lymph nodes was found in 109 of the 180 patients. Of the 71 patients with disagreement between EBUS and MLP at the respective highest echelon, 12 were definitively EBUS+ by histopatholgic proof. pts: patients**Flow chart:** Consecutive patients that match the inclusion criteria of this retrospective trial, i.e. conduct of both EBUS/TBNA and PET/CT before start of treatment in patients with locally advanced non-small cell lung cancer receiving definitive or neoadjuvant radiochemotherapy at the West German Cancer Center. The chart shows diagnostic cross tabulation of the results of EBUS-TBNA (EBUS+ vs. EBUS-) with the results of the cross-validated PET-based multilayer perceptron neural network classifier (MLP+, MLP-) at a sensitivity of 94.5% per lymph node (LN) and per patient. Agreement of both tests in all sampled lymph nodes was found in 109 of the 180 patients. Of the 71 patients with disagreement between EBUS and MLP at the respective highest echelon, 12 were definitively EBUS+ by histopatholgic proof. pts: patients
