# Supplementary figures and images for: Prediction of malignant lymph nodes in NSCLC by machine-learning classifiers using EBUS-TBNA and PET/CT
Source: Sci Rep. 2022 Oct 20;12:17511. doi: 10.1038/s41598-022-21637-y (PMC9584941; doi:10.1038/s41598-022-21637-y)

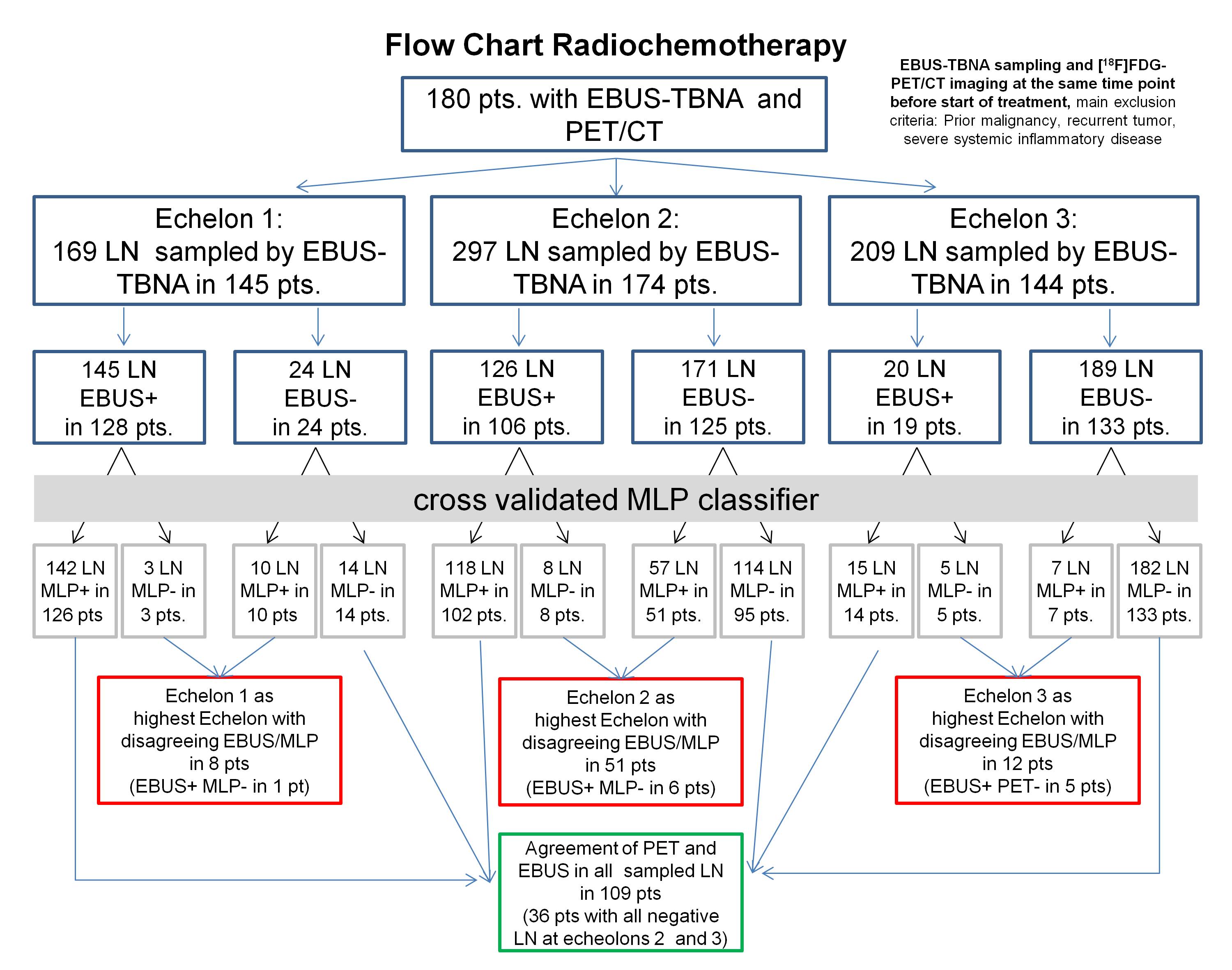

Supplement: Supplementary file 2 — Supplementary Figure 1. [file 41598_2022_21637_MOESM2_ESM.jpg]
